# First report of human infection caused by swine-origin influenza A(H1N2)v virus in mainland China: a confirmed case identified through influenza-like illness surveillance, Kunming (Yunnan), 2025

**DOI:** 10.3389/fpubh.2026.1893536

**Published:** 2026-07-29

**Authors:** Dan Zhao, Siyi Luo, Xiaodie Zhang, Zhipeng Mao, Yang Zhou, Min Dai, Ming Zeng, Xinle Zhang, Lu Chao, Hongmei Pan

**Affiliations:** 1 Department of Acute Infectious Disease Prevention and Control (Emergency Response Office), Kunming Center for Disease Control and Prevention, Kunming, Yunnan, China; 2 Emergency Response Office, Zhaotong Center for Disease Control and Prevention, Zhaotong, Yunnan, China; 3 School of Public Health, Kunming Medical University, Kunming, Yunnan, China

**Keywords:** case report, H1N2v, human infection, influenza-like illness, swine influenza

## Abstract

**Objective:**

This case report details the epidemiological characteristics of the first human case of swine-origin influenza A(H1N2)v infection identified in mainland China, to formulate guidelines for the prevention and control of human swine-origin influenza A (H1N2) virus infections.

**Methods:**

Clinical and epidemiological data of the case were collected through field epidemiological investigation combined with laboratory testing. Specimens were collected from the patient, close contacts, animals subjects (including one deceased pig), and environmental matrices and analyzed to trace the potential infection source and characterize the clinical and epidemiological features.

**Results:**

This study describes a sporadic, isolated case of human infection with swine influenza A(H1N2)v in mainland China, with no evidence of human-to-human transmission. Prior to symptom onset, the patient inhabited a high-risk mixed residential setting with frequent close exposure to domestic livestock and poultry. Specially, his townhouse stood adjacent to his great-uncle’s residence, where swine were raised; the great-uncle also documented the mortality of one pig. But had no contact with human confirmed cases presenting similar clinical manifestations. Testing of the collected specimens confirmed influenza N2 subtype positivity in lung tissue specimens obtained from the deceased pigs, whereas all remaining animal and environmental specimens yielded negative test results. The patient was eventually discharged after clinical recovery. A total of 30 close contacts completed self-health monitoring and throat swab screening. No influenza-like illness developed among these individuals throughout the monitoring period, and all throat swab specimens yielded negative test results. No secondary cases were detected during medical monitoring, and targeted environmental disinfection was implemented accordingly.

**Conclusion:**

The likely source of infection for the reported case was identified as deceased pigs harboring N2-subtype influenza A virus. Therefore, enhanced surveillance of novel influenza subtypes such as A(H1N2)v and improved swine influenza vaccination are crucial for the targeted prevention and control of human infections by these emerging viruses.

## 1 Introduction

Influenza viruses are members of the *Orthomyxoviridae* family and classified as negative-sense, single-stranded RNA viruses. They are divided into four major genera: types A, B, C and D (1). As a critical zoonotic pathogen, influenza A virus triggers severe epidemic diseases in both humans and livestock, and has been responsible for four major global influenza pandemics throughout history (2), including the 1918 Spanish flu (H1N1), 1957 Asian flu (H2N2), 1968 Hong Kong flu (H3N2), and the 2009 swine-origin pandemic H1N1 (pH1N1) (3).

Swine influenza virus (SIV) is classified under influenza A virus. The predominant SIV subtypes circulating in pig herds worldwide are currently H1N1, H1N2 and H3N2 (4). Meanwhile, rare subtypes including H3N1 (5), H1N7 (6), H4N6 (7), highly pathogenic avian influenza (HPAI) H5N1 (8) and H9N2 (9) have also been isolated from swine populations. The H1N2 subtype was first identified in pig herds in Japan in 1978, arising from genetic reassortment between human H1N1 and H3N2 influenza viruses. Its neuraminidase (NA) gene is derived from human H3N2 viruses, whereas the remaining seven gene segments originate from classical H1N1 SIV, defining it as a dual-reassortant strain (10, 11). In 2004, the first domestic outbreak of H1N2 swine influenza was documented in Zhejiang Province, China. The isolated strain exhibited high genetic similarity to the Japanese prototype strain and further caused a widespread swine influenza epidemic in 2006 (12). Furthermore, novel triple-reassortant H1N2 influenza viruses were recovered from pigs in southern China and South Korea during 2011–2012 (13, 14). Notably, after the 2009 pandemic H1N1 virus spilled over from humans to pigs (15), extensive genetic reassortment took place among SIVs with distinct subtypes and genotypes. This evolutionary change has dramatically accelerated the emergence of novel reassortant H1N2 variants.

Sporadic human infections with swine influenza virus (SIV) have been continuously reported globally (16–25). A number of human cases infected with swine-origin H1N2 influenza virus have also been documented in previous studies (21, 26–30). For cases linked to close exposure to infected swine, the suffix “v” (variant) is added to the virus subtype nomenclature to denote its swine origin (31). To strengthen influenza prevention and surveillance, Kunming city, Yunnan Province, has conducted routine influenza-like illness (ILI) monitoring since 2017 in accordance with the *National Technical Guidelines for Influenza Surveillance* (32). Sentinel hospitals collect approximately 20 ILI specimens routinely each week for laboratory testing as part of standard procedures. The present investigation originated from one such routine surveillance activities, swine H1N2 influenza virus was identified in human throat swab samples. This represents the first detection of influenza A(H1N2)v in human respiratory specimens across mainland China.

Centers for Disease Control and Prevention (CDCs) at the provincial, municipal and county levels promptly initiated field epidemiological investigations and instituted comprehensive prevention and control measures. To furnish scientific evidence and theoretical support for the emergency management of analogous outbreaks in the future, this article systematically summarizes and analyzes the epidemiological characteristics of the present case.

## 2 Methods

### 2.1 Clinical information

The patient was admitted to Kunming Children’s Hospital, a national sentinel hospital within the Yunnan Provincial Influenza Surveillance Network. As part of the influenza-like illness (ILI) surveillance program, the hospital collected throat swab samples from ILI patients and sent them to the Yunnan Center for Disease Control and Prevention (YNCDC). All specimens were tested for influenza viruses using real-time reverse transcription polymerase chain reaction (RT-qPCR). To further characterize the clinical manifestations of this case, we collaborated with the admitting hospital to collect detailed clinical data, including medical records, laboratory test results, clinical diagnoses, and medication histories.

### 2.2 Field epidemiological investigation

This epidemiological investigation was carried out in strict accordance with the *Technical Guidelines for Prevention and Control of Human Infection with Animal-Origin Influenza* (32). Issued by the General Office of the National Health Commission of China on July 15, 2021, this guideline strengthens the scientific and standardized prevention and control of human infections with animal-origin influenza in China. In strict accordance with the guideline requirements, a joint field investigation was conducted by CDCs at the provincial, municipal, and county levels. Detailed epidemiological data were collected through in-depth interviews with the patient’s guardian, covering demographic characteristics, living environments, clinical disease progression, daily activities trajectories, animal exposure history, and close contact information. These data were analyzed to trace potential infection sources and clarify the transmission chain of the case.

### 2.3 Field specimen collection and laboratory testing

On January 26, a throat swab sample was collected from the index patient by the hospital. A joint field epidemiological investigation was conducted from January 30 to February 1 by the YNCDC, Kunming Municipal Center for Disease Control and Prevention (KMCDC), Zhaotong Municipal Center for Disease Control and Prevention (ZHTCDC), and Zhenxiong County Center for Disease Control and Prevention (ZHXCDC). Multiple categories of specimens were gathered throughout this investigation, covering close contacts, the treating hospital, the patient’s residence, associated environmental sites, and the local slaughterhouse.

Specifically, throat swabs were sampled from close contacts. Environmental swabs and sewage samples were simultaneously collected from the hospital, residence, and slaughterhouse. For domestic livestock and poultry, nasal, oropharyngeal and cloacal swabs, as well as fecal and whole blood samples, were collected from pigs, chickens, geese and pigeons. Supplementary food and drinking water samples were taken from chicken breeding areas, and surface swabs were obtained from the feeding tools and cages of pigs and pigeons.

Furthermore, clinical and post-mortem tissues and fluids, including whole blood, pleural effusion, bronchoalveolar lavage fluid, lung parenchyma and tracheal tissue, were harvested from deceased pig carcass. Detailed information on specimen collection is presented in Table 1. All specimens were tested for influenza viruses using RT-qPCR.

**Table 1:** Sampling and influenza nucleic acid detection results for human, animal, and environmental specimens associated with the novel Influenza A(H1N2)v case.

Sampling date	Source item	Specimen type	No. of samples	Result
Jan 30, 2026	Close contacts in Kunming	Throat swabs	17	Negative
	Environmental specimens from hospital wards	Environmental surface swabs	11	Negative
Jan 31, 2026	Close contacts at the residence	Throat swabs	13	Negative
	Patient’s room and toys	Environmental surface swabs	6	Negative
	Livestock and poultry raised by the patient’s grandfather (pigs, chickens, geese, pigeons)	Nasal, throat, and cloacal swabs	7	Negative
	Livestock and poultry raised by the patient’s grandfather (pigs, chickens,)	Feces	3	Negative
	Poultry (chickens) raised by patient’s grandfather	Food and drinking water	2	Negative
	Feeding tools for pigs and cages for pigeons raised by the patient’s grandfather	Surface swabs	2	Negative
	Livestock (pigs) raised by the patient’s great-uncle	Nasopharyngeal swabs	3	Negative
	Livestock (pigs) raised by the patient’s great-uncle	Feces	2	Negative
	Chopping boards, roasting stoves, and sewage at the slaughterhouse	Chopping board surface swabs, sewage	3	Negative
Feb 1, 2026	Three pigs raised by the patient’s grandfather	Oropharyngeal and cloacal swabs	3	Negative
		Whole blood	3	Negative
	Three pigs raised by the patient’s great-uncle	Oropharyngeal and cloacal swabs	3	Negative
		Whole blood	3	Negative
	One dead pig^#^	Whole blood	1	Negative
		Pleural effusion	2	Negative
		Bronchoalveolar lavage fluid	1	Flu A Positive (CT: 35.43–38.33)
		Lung tissue	2	Flu A Positive (CT: 35.43–38.33)
		Trachea	1	Negative
	Pigs at the slaughterhouse	Pig whole blood	2	Negative
	Cured meat prepared from pigs of the patient’s grandfather	Surface swab of cured meat	1	Negative

^#^Specimens were collected from one dead pig raised by the patient’s great-uncle. Bronchoalveolar lavage fluid and lung tissue tested positive, while other specimens yielded negative results.

## 3 Results

### 3.1 Case presentation

The patient was a 2-year-and-10-month-old male child (body weight: 16 kg), residing in a scattered settlement in Poji Town, Zhenxiong County, Zhaotong City, Yunnan Province. His parents were migrant workers, and the child lived with his paternal grandparents. No contact with neighboring residents was documented prior to symptom onset. Per caregiver reports, the child was generally in good health with no notable underlying diseases. He had no history of drug or food allergies, no recent exposure to contagious pathogens, and no familial genetic diseases.

### 3.2 History of present illness

As the patient’s parents were absent, the child was brought to Poji Central Health Center (Zhenxiong County) by his aunt. According to the aunt’s account, the patient developed an acute, unprovoked cough on January 20. The cough presented in paroxysmal attacks, with 3–4 bouts per episode and over 10 episodes per day, predominantly occurring in the morning and at night. Concurrent clinical signs included audible rhonchi, mild rhinorrhea, and nasal congestion. The patient also developed fever, with a peak temperature of 38.9 °C recorded between 14:00 and 15:00, which resolved after ibuprofen administration. No gastrointestinal manifestations, including abdominal pain, vomiting or diarrhea, were reported throughout the course of illness.

On January 22, 2026, the patient attended the outpatient clinic at the same health center. Chest CT and complete blood count (CBC) tests confirmed diagnoses of acute upper respiratory tract infection and acute bronchitis. Multiple pediatric medications were administered, including Pediatric Paracetamol, Artificial Cow bezoar and Chlorphenamine Maleate Granules, Pediatric Yanbian Granules, Pediatric Kechuanling Granules and Ibuprofen Suspension, but clinical response was poor.

On January 23, the patient returned to the health center and received an intramuscular injection of Compound Aminophenazone and Barbital Injection plus oral Cefaclor for Suspension, yet symptoms remained unrelieved.

On January 24, the patient presented to Jingan Clinic accompanied by his grandmother, and herpangina was diagnosed. A combined rectal injection of 1 mL Compound Aminophenazone and Barbital Injection and 0.1 g Ribavirin Injection was administered. Meanwhile oral medications including Pediatric Qingyan Granules, Amoxicillin Granules and Antiviral Oral Liquid were prescribed. As a result, his clinical symptoms did not ameliorate, with continuous cough and recurrent fever remaining unresolved.

On January 25, the patient was transferred to Kunming for further medical evaluation and treatment. He presented to the outpatient clinic of Kunming Children’s Hospital on January 26. Given his prolonged fever and rapid clinical deterioration, he was subsequently hospitalized with a confirmed diagnosis of bronchopneumonia and infectious fever.

Upon admission, physical examination showed the patient’s body temperature was 36.5 °C and respiratory rate was 28 breaths per minute. The patient remained conscious but presented with a poor general status and decreased responsiveness. Physical findings included pharyngeal congestion, grade II tonsillar hyperemia, bilateral coarse breath sounds, and audible rhonchi.

Upon admission, laboratory and imaging results were summarized as follows: the white blood cell count was 6.36 × 109/L, with a neutrophil proportion of 44.2% and a lymphocyte proportion of 43.9%. The hemoglobin level was 120 g/L, and the C-reactive protein concentration was elevated at 50.26 mg/L. Respiratory pathogen screening yielded positive results for influenza A virus. Chest CT scans revealed increased and blurred pulmonary markings, while no abnormal lesions were observed in the heart or diaphragm. Sputum culture isolated *Haemophilus* species, which was preliminarily identified as *Haemophilus influenzae* with a 70% detection probability.

**Figure 1:**
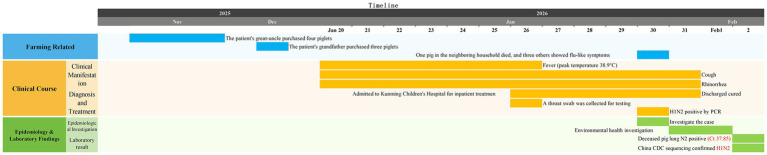
Timeline of the patient’s suspected exposure, clinical treatment, and epidemiological investigation.

Upon admission, the patient received combined anti-infective therapy with cefoperazone-sulbactam, anti-influenza treatment with oseltamivir, ambroxol for expectoration, and budesonide nebulization to alleviate airway inflammation and achieve bronchodilation. A low-grade fever (peak temperature: 38 °C) developed on the day of admission, which resolved following physical cooling without subsequent recurrence. Thereafter, the patient’s cough gradually diminished, lung rhonchi disappeared, and his overall clinical condition improved significantly. The child achieved complete recovery and was discharged on January 31 (Figure 1).

### 3.3 Environmental and exposure history

The patient was a long-term resident in a rural area approximately 4 kilometers from the town center of Poji Town, Zhenxiong County, around 20 kilometers away from the county seat. The household was located in a shared courtyard compound inhabited by three families: the patient’s grandfather, a neighboring household, and the patient’s great-uncle. The patient lived with his grandfather, and the family raised various livestock and poultry, including pigs, chickens and pigeons.

**Figure 2:**
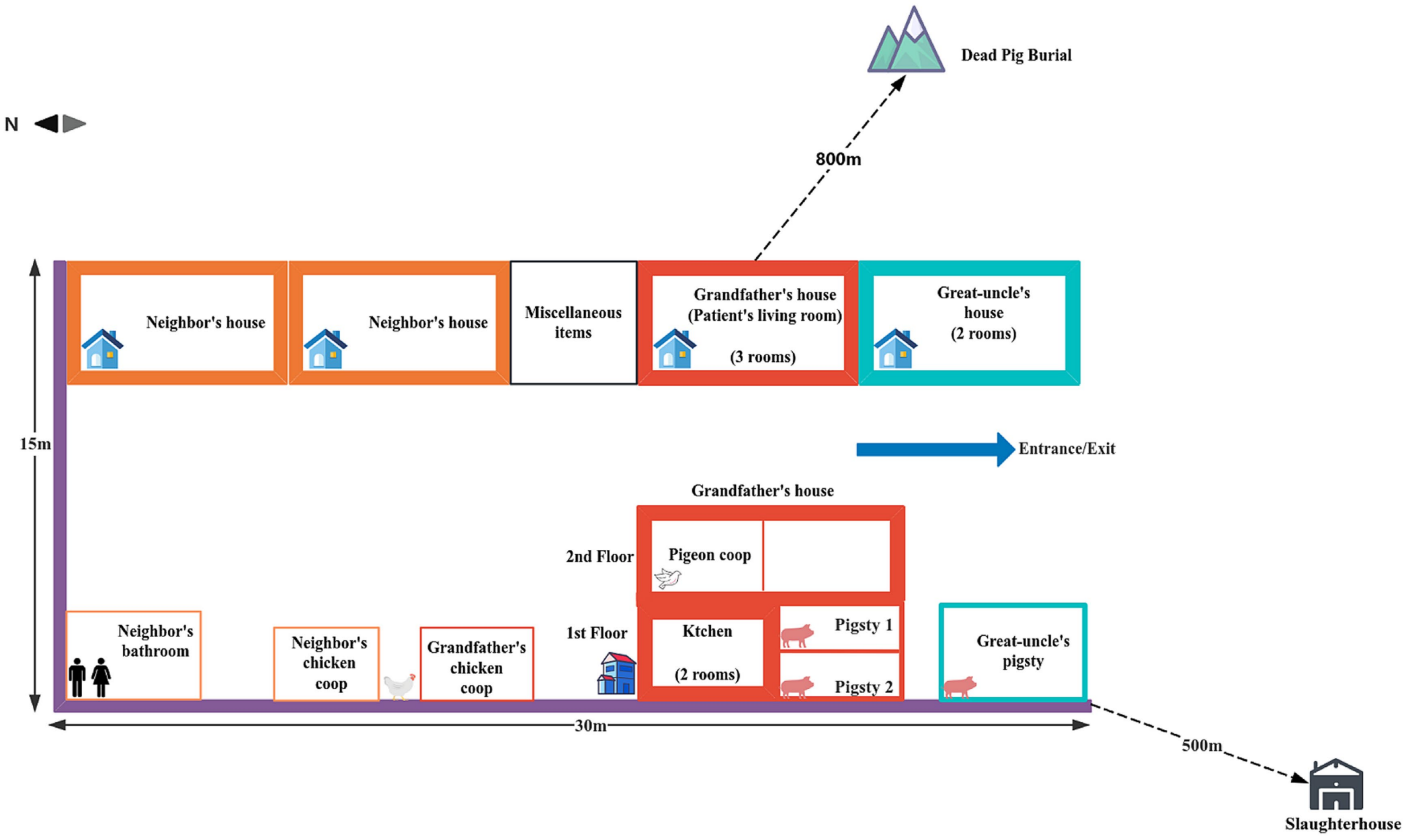
Schematic illustration of the patient’s living environment and potential surrounding infection sources.

The patient’s sleeping quarters were situated in a three-room ground-floor stone-building. He occupied a 15-square-meter inner room with substandard indoor sanitation. Directly opposite his room stood a two-story brick-concrete main residential building that was adjacent to pigsties and chicken coops. On the ground floor of the main building, two pigsties were located on the left side, with a kitchen and living areas at the front. The second floor was used pigeon rearing, while free-range chickens were raised in the courtyard. The entire household compound was shared by three families. Notably, the great-uncle’s pigsty was situated in a courtyard near the entrance, adjacent to the grandfather’s pigsty. In an adjacent to the courtyard, a neighboring household raised chickens in wire-mesh coops next to a latrine. Overall, the residential environment exhibited poor sanitary conditions (Figure 2). The patient had frequent direct contact with free-range chickens and scattered feces during daily activities, including commuting between his sleeping quarters and the main living area and playing in the courtyard. Additionally, the close spatial proximity between the kitchen and the pigsties enabled regular contact between the patient and livestock. Notably, the residence’s close proximity to various domestic animals created persistent exposure risks to zoonotic pathogens.

### 3.4 Livestock and poultry rearing

Around the Winter Solstice (December 21), the patient’s grandfather sent a house-raised pig to a local slaughterhouse near a stone bridge in Wafang Group, Liguanying Village, Poji Town, for slaughter. The slaughterhouse was approximately 500 meters away from the family’s residence. After the slaughtering, the pigsties underwent preliminary disinfection via quicklime spraying. Part of the pork was cured for domestic consumption. Three to 4 days after the slaughter, three piglets were purchased from a mobile itinerant vendor, with the exact source of the piglets remains untraceable.

The patient’s great-uncle owned five pigsties, with pigs obtained in two batches. One pig was purchased approximated November 2024 and later slaughtered for sale. Four piglets were acquired around November 2025. On January 30, one of these four piglets died. An insurance claim was submitted, and the carcass was buried on-site. Of the remaining three pigs, two developed symptoms, including coughing and diarrhea.

### 3.5 Laboratory testing

On January 30, field epidemiological investigations were conducted by YNCDC and KMCDC, and throat swab samples were collected from the patient for influenza A virus detection via RT-qPCR. All testing were negative. Meanwhile, archived clinical specimens preserved by the admitting hospital were retrieved and re-tested by YNCDC on the same day, which also yielded negative RT-qPCR results for influenza A virus (Figure 1).

At 14:40 on January 31, the YNCDC sequenced the patient’s oropharyngeal swab and acquired the complete viral genome. Raw data were submitted to the National Influenza Center by 15:00. Bioinformatic analysis of raw reads confirmed the presence of a full-length genome influenza A(H1N2) genome. Phylogenetic analysis showed that the virus originated from swine H1N1, H1N2, and H3N2 lineages, and all eight segments shared 95.17–98.53% nucleotide homology with endemic swine influenza strains in China. Representing the first human case of infection with this novel subtype reported in China. The specimen was submitted to the Chinese Center for Disease Control and Prevention (China CDC) for further verification on January 31. On February 2, whole genome sequencing by the China CDC ultimately verified the isolate as swine A(H1N2)v.

### 3.6 Contact tracing and medical observation

In accordance with the *Technical Guidelines for the Prevention and Control of Human Infection with Animal-Origin Influenza*, close contacts were defined as individuals who had direct exposure to the index patient without personal protective equipment (PPE) during the period from January 19 to January 31. A total of 30 close contacts were identified, consisting of family members, neighboring residents, and fellow travelers. None of these individuals developed A(H1N2)v associated symptoms or other respiratory illnesses within 10 days after their last exposure to the patient. Throat swab samples were collected from all close contacts and subjected to influenza virus detection via RT-qPCR, with all test results returning negative.

### 3.7 Tracing investigation

A total of 91 specimens were collected from multiple sources during the field and environmental epidemiological investigations.

Specifically, on January 30, the YNCDC and KMCDC conducted an epidemiological investigation into the patient’s travel history and activity trajectories in Kunming, collecting 28 specimens for nucleic acid testing. These included 17 throat swabs from close contacts and 11 environmental specimens from the treating hospital.

On January 31, a joint investigation team comprising the YNCDC, ZHTCDC, and ZHXCDC conducted an on-site investigation in Poji Town, Zhenxiong County, and collected 41 specimens.

These comprised 13 throat swabs from close contacts at the patient’s residence; 10 nasal, throat, and cloacal swabs from livestock and poultry (pigs, chickens, geese, and pigeons); 2 food and drinking water samples from chickens; 5 fecal specimens from pigs and chickens; and 11 environmental swabs and sewage samples from key surveillance sites.

On February 1, the joint investigation team under the guidance of the Zhenxiong County Animal Health Supervision Institute collected 22 additional specimens in Poji Town. These included 3 oropharyngeal and cloacal swabs, and 3 whole blood samples from pigs raised by the patient’s grandfather; 3 oropharyngeal and cloacal swabs, and 3 whole blood samples from pigs raised by the patient’s great-uncle; 1 whole blood sample, 2 pleural effusion samples, 1 bronchoalveolar lavage fluid (BALF) sample, 2 lung tissue samples, and 1 trachea sample harvested from a dead pig; 2 pig whole blood samples from a local slaughterhouse; and 1 surface swab from cured meat prepared from pigs raised by the patient’s grandfather.

Nucleic acid testing for the influenza A virus was subsequently performed on all 91 specimens using RT-qPCR.

Following verification by the YNCDC, one BALF sample and two lung tissue samples tested positive for influenza A, with Ct values ranging from 35.43 to 38.33. Subtypes was further conducted on the lung tissue specimen with the lowest Ct value (35.43) among the positive samples. This specimen was confirmed positive for the N2 subtype with a Ct value of 37.85, while all remaining specimens tested negative. Detailed sampling and testing results are summarized in Table 1.

### 3.8 Focus disposal and surveillance

The dead pig raised by the patient’s great-uncle was incinerated in accordance with standardized biohazard harmless disposal protocols. From February 1 to 3, daily terminal disinfection was implemented at the patient’s living premises, surrounding environments, and poultry activity areas using handheld sprayers, covering a total area of approximately 600 square meters. Enhanced respiratory disease surveillance was continuously carried out in Kunming and Zhaotong. As of February 10, no secondary cases of infection had been identified or reported.

## 4 Discussion

Through this established influenza surveillance system, a human case infected with the variant swine influenza A(H1N2) virus was successfully identified. This represents the first detection of such a case via local ILI surveillance, underscoring the critical role of ILI monitoring in early warning, epidemic tracking, and timely identification of emerging or reassortant influenza strains (33, 34).

Sustained surveillance of patients with influenza-like symptoms enables timely recognition of abnormal increases in disease activity, facilitates early risk alert before epidemic escalation. This mechanism affords public health authorities sufficient lead time to trace transmission chains rapidly and evaluate the magnitude and public health impact of potential outbreaks (35). In addition, systematic specimen collection and etiological testing support real-time monitoring of influenza subtype distribution and viral genetic evolution. Such monitoring is critical for the early detection of novel or reassortant variants, assessment of vaccine matching, and prediction of epidemic trends, thereby providing a scientific foundation for vaccine optimization as well as the formulation and adjustment of targeted prevention and control strategies (36).

In the present case, the patient’s main clinical manifestations were fever and cough. The patient had a concurrent *Haemophilus influenzae* co-infection, resulting in a combined multiple-pathogen pneumonia upon admission. With standardized treatment, the child recovered fully and was discharged 4 days after hospitalization. For comparison, a 5-year-old A(H1N2)v case reported in Taiwan, China (27) only presented with cough and fever, while a patient under 18 years of age in the United States (28), developed a wider spectrum of respiratory and systemic symptoms, including fever, cough, sore throat, myalgia, headache, dyspnea, diarrhea, nausea, dizziness, and somnolence, without requiring hospital admission. A 16-year-old female patient in Brazil (30) suffered from fever, cough, sore throat, chest pain, and myalgia. Overall, the clinical features of A(H1N2)v infection are dominated by classic influenza-like illnesses such as fever, cough, and headache, which are highly consistent with the symptom profiles of H1N1v and H3N2v variant influenza infections (24, 25). These clinical manifestations are largely non-specific and resemble those of uncomplicated seasonal influenza (37). Current epidemiological evidence indicates that infections caused by H3N2v and H1N2v are generally milder than H1N1v infection, with lower hospitalization risks and case fatality rates (28, 38). Nevertheless, continuous vigilance is still warranted. A(H1N2)v variants and other swine-origin influenza viruses are persistently detected in swine populations (39). Additionally, humans generally possess low level of pre-existing immunity against swine-origin A(H1N2)v influenza strains, raising substantial concerns regarding the zoonotic potential and transmissibility of these virus in humans. If the virus acquires efficient human-to-human transmission capacity that differs from currently circulating seasonal human influenza viruses, it may trigger widespread human epidemics.

No secondary cases were detected throughout the investigation, and no evidence of human-to-human transmissibility was identified. This observation aligns with previous research demonstrating that swine-origin variant influenza viruses generally exhibit poor transmissibility among humans, with zoonotic spillover from pigs serving as the predominant infection route (25, 27). Infections caused by swine-origin influenza A viruses occur mostly as sporadic individual cases, and clustered outbreaks are uncommon. Where small clusters do emerge, they are typically linked to shared zoonotic exposure-such as attendance at agricultural fairs and swine exhibitions in the United States-rather than continuous human-to-human transmission (40, 41).

Epidemiological investigations confirmed that the patient had frequent close contact with domestic livestock and poultry. Specifically, the patient resided in a townhouse adjacent to the dwelling of his great-uncle, who raised pigs and reported the death of one of them. Although two of the remaining pigs developed symptoms such as coughing and diarrhea, they tested negative for influenza. Furthermore, the great-uncle’s pigsty was situated in a courtyard near the entrance. Crucially, an N2-subtype influenza virus was identified in lung tissue specimens from the deceased pig, strongly suggesting a plausible exposure route. Collectively, these investigations confirmed a high-risk mixed-residence environment, where humans, poultry, and pigs were housed within the same compound. Such frequent cross-exposure between poultry and livestock further significantly increases the risk of genomic reassortment among distinct influenza virus strains (2, 42). Overall, close and frequent contact with pigs markedly amplifies the risk of zoonotic transmission of swine influenza to human populations (43, 44).

Although the influenza N2 subtype was detected in lung tissue specimens from deceased pigs, all other environmental and biological samples tested negative by RT-qPCR, most likely due to delayed sampling. Viral particles released into the environment are short-lived and readily undergo rapid inactivation and degradation under natural environmental stressors such as desiccation, high ambient temperatures, and ultraviolet radiation (45). Accordingly, negative environmental testing cannot exclude temporary environmental contamination at the time of exposure, yet it indicates that persistent viral accumulation in the surrounding environment was not required for this infection to occur. Combined with comprehensive epidemiological evidence, our findings support that exposure within a shared human-animal living environment may have contributed to viral infection, although the exact transmission route could not be definitively identified.

Integrated with the patient’s living environment and epidemiological exposure history, the available evidence suggests that the N2-positive deceased pig by the patient’s great-uncle at the neighboring residence were a plausible source of exposure. To mitigate such public health risks, strict livestock segregation is strongly recommended. Poultry and swine should be raised in enclosed, independent facilities that are physically separated from residential areas. This targeted intervention is critical to reduce interspecies viral reassortment and enhance overall household biosecurity. Currently, vaccination against swine influenza viruses (SIVs) is not widely implemented for prevention and control across China. Individuals engaged in swine-related occupations routinely maintain close contact with pig herds during daily work. Indeed, previous studies have documented elevated seroprevalence of swine influenza antibodies among these high-risk occupational groups (46). Reliance on passive surveillance alone is insufficient to fully curb viral transmission. Accordingly, we recommend that health authorities scale up public education and outreach campaigns to promote seasonal influenza vaccination among occupationally exposed populations. Establishing an immune barrier among this high-risk demographic can not only protect the occupational health of frontline workers but also mitigate the risks of cross-species viral transmission and genetic reassortment at the source, thereby improving overall biosafety and influenza prevention capacity.

Since the first isolation of the H1N2 subtype SIV, 16 distinct genotypes have been identified in mainland China. The introduction and widespread circulation of the 2009 pandemic H1N1 virus in swine populations have markedly accelerated the emergence of reassortant SIV strains, rendering the patterns of viral genetic reassortment increasingly intricate and diverse (47, 48).

Swine act as one of the most important natural reservoirs for influenza A viruses. Although no targeted vaccine is currently available to prevent human infection with the A(H1N2)v variant, seasonal influenza vaccination remains an essential preventive strategy (49). While this investigation found no evidence of human-to-human transmissibility, the virus poses a potential pandemic threat should it acquire the capacity for sustained transmission. Crucially, while routine vaccination may not offer specific protection against A(H1N2)v, it confers cross-protection against circulating strains and reduces the overall burden of seasonal influenza.

Consequently, research institutions should prioritize the development of multivalent swine influenza vaccines. In parallel, public health and agricultural authorities must strengthen vaccination coverage in livestock to curb zoonotic transmission at the source (50, 51).

This study highlights a clear association between traditional backyard farming practices and the elevated risks of swine influenza virus reassortment and cross-species transmission at the poultry–swine–human interface. In these rural rearing settings, humans, poultry, and pigs frequently cohabitate within confined, poorly segregated living spaces. Nevertheless, substantial information silos and response delays currently exist between human disease surveillance systems and animal husbandry pathogen monitoring programs.

Consequently, establishing a bidirectional influenza information-sharing mechanism under the One Health framework is urgently needed. Such efforts require closer collaboration and standardized collaborative mechanisms with animal health authorities. Routine pathogen surveillance carried out by veterinary agencies on farms, in live poultry markets, and at slaughterhouses can provide critical early warning signals for emerging and reassortant influenza viruses. Meanwhile, epidemiological traceback data from human zoonotic cases help guide targeted, precise prevention and control strategies at the animal source.

The negative result of the archived specimen (collected on January 26) retested by the YNCDC on January 30 was likely a false negative. This testing discrepancy can be primarily attributed to two factors. First, the sample was collected at the very early stage of symptom onset. Considering the highly dynamic pattern of viral shedding, the viral load in the patient’s upper respiratory tract had not peaked at the time and was probably below the limit of detection (LOD) of the RT-qPCR assay. Secondly, suboptimal sampling sites with lower viral concentrations or an insufficient collection of infected epithelial cells may further contributed to false-negative results. In comparison, the Next-Generation Sequencing (NGS) employed in this study exhibited higher analytical sensitivity, enabling the identification of trace viral nucleic acids. Benefiting from such high sensitivity, the full genome was successfully recovered, which confirmed the influenza infection despite the initial negative RT-qPCR findings.

Accordingly, an integrated collaborative model focused on *animal-source intervention to block zoonotic spillover* is essential to achieve effective upstream containment and mitigate the public health threats posed by novel influenza viruses.

The source of infection for China’s first human case of novel influenza A(H1N2)v is the most plausibly deceased pigs that tested positive for N2-subtype influenza A virus. Therefore, enhanced surveillance for A(H1N2)v and other swine-origin influenza variants, scaled-up swine influenza vaccination, strengthened cross-sector collaboration, and improvements to traditional mixed-farming practices are urgently required to enable the scientific prevention and targeted control of zoonotic influenza threats.

This study elucidates the transmission dynamics of A(H1N2)v, although several limitations remain. First, complete genomic sequences of the patient’s A(H1N2)v from China CDC testing were unavailable. Furthermore, the swine lung tissue specimens exhibited relatively high Ct values, which precluded successful sequencing. While comparisons with global reference sequences were theoretically possible, the lack of viral sequences from the patient limited the comprehensive evaluation of viral evolution. Additionally, relying solely on PCR without supplementary serological testing restricted the depth of the diagnostic evaluation. Future research should optimize sequencing protocols for low-viral-load samples and strengthen cross-sector data sharing under the One Health framework to further clarify the zoonotic transmission pathways of A(H1N2)v.

## Data Availability

All data relevant to this case report are presented within the manuscript. Further information is available from the corresponding author upon reasonable request.
